# An Overview of Eco-Driving Theory, Capability Evaluation, and Training Applications

**DOI:** 10.3390/s21196547

**Published:** 2021-09-30

**Authors:** Nan Xu, Xiaohan Li, Qiao Liu, Di Zhao

**Affiliations:** State Key Laboratory of Automotive Simulation and Control, Jilin University, Changchun 130025, China; nanxu@jlu.edu.cn (N.X.); xiaohan19@mails.jlu.edu.cn (X.L.); liuqiao20@mails.jlu.edu.cn (Q.L.)

**Keywords:** eco-driving, energy consumption, evaluation, eco-driving training, feedback devices

## Abstract

Constrained by traditional fuel-saving technologies that have almost reached the limit of fuel-saving potential, the difficulty in changing urban congestion, and the low market penetration rate of new energy vehicles, in the short term, eco-driving seems to be an effective way to achieve energy-saving and emissions reduction in the transportation industry. This paper reviews the energy-saving theory and technology of eco-driving, eco-driving capability evaluation, and the practical application of eco-driving, and points out some limitations of previous studies. Specifically, the research on eco-driving theory mostly focuses on a single vehicle in a single scene, and there is a lack of eco-driving research for fleets or regions. In addition, the parameters used to evaluate eco-driving capabilities mainly focus on speed, acceleration, and fuel consumption, but external factors that are not related to the driver will affect these parameters, making the evaluation results unreasonable. Fortunately, vehicle big data and the Internet of Vehicles (V2I) provides an information basis for solving regional eco-driving, and it also provides a data basis for the study of data-driven methods for the fair evaluation of eco-driving. In general, the development of new technologies provides new ideas for solving some problems in the field of eco-driving.

## 1. Introduction

Energy issues and global climate change issues have received extensive attention from countries all over the world. In September 2015, the United Nations (UN) formulated 17 Sustainable Development Goals. In order to achieve these goals, various departments need more policies and to make more effort, especially the energy sector, because energy is a key contributor to all development activities [1]. In December of the same year, the Paris Agreement was adopted at the Paris Climate Change Conference to deal with global warming [2]. As a major energy consumer and a major source of greenhouse gases, the transportation industry, especially in urban areas, is the industry with the highest energy consumption [3]. The transportation industry should contribute to solving the above-mentioned problems.

**Figure 1:**
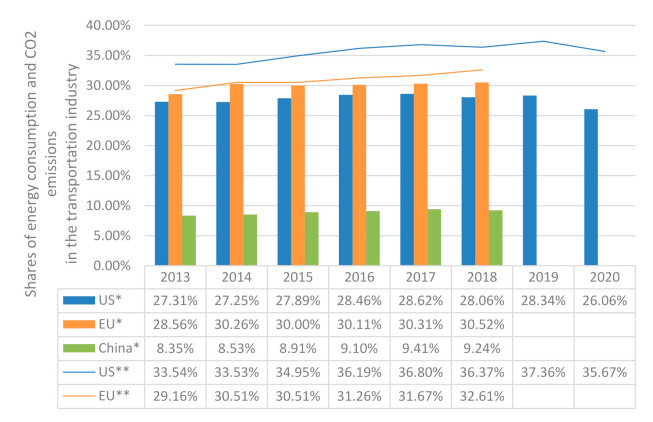
Shares of energy consumption and CO_2_ emissions in the transportation industry in the United States, China, and the European Union. Symbols for this figure: * shares of energy consumption, ** shares of CO_2_ emissions. Vacant data means not found.

In the United States (U.S.), the transportation industry in 2020 accounted for approximately 26% of total U.S. energy needs and 58% of petroleum consumption [4]. In China, the transportation industry also consumed a lot of petroleum resources (about 46%) in 2018 [5]. As shown in Figure 1, from 2013 to 2018, the energy consumption of the transportation industry in the United States and the European Union (EU) remained at 25%–30% [4,6]. Even as a developing country, China’s energy consumption in the transportation industry also accounted for close to 10% [5]. Huge energy consumption has brought a lot of carbon dioxide emissions. Since 2014, the share of carbon dioxide emissions in the transportation industry in the United States and the European Union has exceeded 30%. The increase in greenhouse gas emissions has brought about global warming. In order to achieve the global carbon neutrality goal and Sustainable Development Goals as soon as possible, the transportation industry’s key role in reducing energy consumption and greenhouse environmental emissions cannot be ignored [1,2,3].

Vehicle energy consumption is the result of the comprehensive action of drivers, vehicles, scenarios, and weather. Reducing vehicle energy consumption can be considered from the following aspects: (1) vehicles. Adopting new engine technology and vehicle technology can improve fuel economy by 4–8% and 2–8%, respectively [7]. For example, the use of new tire materials and more streamlined vehicle styling (each reduction in rolling resistance of 5% to 7% increases fuel efficiency by 1%, and air resistance is reduced from 0.08 to 0.06, which is expected to save 8.6% of fuel [8]). (2) Scenario. This includes roads and traffic. Improving the road traffic environment and reducing traffic congestion is an effective way to reduce energy consumption and emissions. Choosing a longer but more unobstructed route or choosing to travel during a less crowded time can effectively reduce energy consumption and emissions [9,10]. In addition, traffic lights [1,11,12] and road slope [13] also have an impact on energy consumption. (3) Weather and temperature. Low temperature [14,15] and bad weather [16,17] can increase energy consumption. Within a certain temperature range, energy consumption will decrease as the temperature rises. Bart et al. [15] showed that for every 1 ℃ decrease in temperature, fuel consumption increases by 0.38% ± 0.0079%, and rainfall may have an impact on energy consumption because rainfall increases rolling resistance and wind resistance [17].

**Figure 2:**
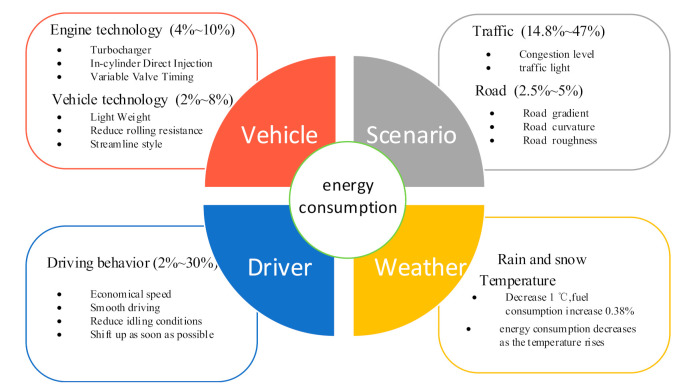
The primary factors affecting vehicle energy consumption.

However, eco-driving seems to be an effective way to achieve energy saving and emissions reduction in the short term [18]. For internal combustion engine (ICE) vehicles, the difference in fuel consumption due to driving style can reach 15%–25% [19]. Since hybrid vehicles (HV) and electric vehicles (EV) can recuperate the kinetic energy of the moving vehicle during braking, their energy consumption is more sensitive to driving behavior than ICE vehicles [20,21,22]. A few studies have indicated that the difference in fuel consumption due to different driving behaviors can reach 50% for hybrid vehicles [21] and the impact of eco-driving on the energy consumption of electric vehicles can reach approximately 25% [22]. As shown in Figure 2, driver factors should be given priority among the primary factors affecting vehicle energy consumption, and eco-driving has great energy-saving potential.

Eco-driving in a broad sense includes vehicle purchase and maintenance, travel decisions, and driving behavior [23]. With the in-depth research of autonomous driving technology, the meaning of eco-driving also includes the control technology of energy-saving driving (such as energy management strategy) that is achieved by optimizing the working point of the engine and motor at the level of vehicle control algorithms. In this study, eco-driving is narrowly defined as without changing the existing vehicle power structure, through training or in-vehicle devices, changing the driver’s bad driving habits to achieve energy-saving driving. The object of this article is human-oriented eco-driving.

**Figure 3:**
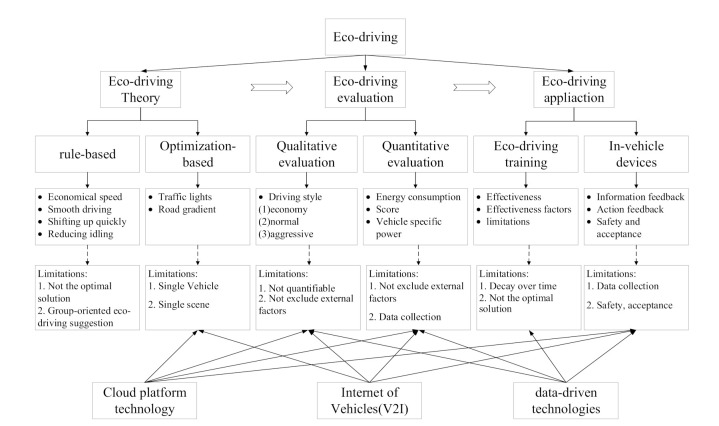
The overall framework of this paper.

This paper reviews and summarizes the main literature in the field of eco-driving from the three progressive levels of theory–evaluation–application. Combined with the current development of artificial intelligence technologies such as vehicle big data, V2I, and cloud computing platforms, it points out the opportunities and challenges faced by eco-driving. Specifically, as shown in Figure 3, Section 2 explains the eco-driving theory from two perspectives: rule based and optimization based. Section 3 reviews the eco-driving capability evaluation method, and it is divided into qualitative evaluation and quantitative evaluation. Section 4 summarizes the effectiveness and limitations of eco-driving training and in-vehicle feedback devices. Finally, in Section 5, the opportunities and challenges of eco-driving are proposed.

## 2. Eco-Driving Theory

This section reviews the research on eco-driving theory, which is to study energy-saving mechanisms and energy-saving driving methods at the theoretical level. According to the different sources of energy-saving driving strategies, it mainly includes rule-based and theory-based eco-driving strategies.

### 2.1. Rule-Based Eco-Driving Theory

**Figure 4:**
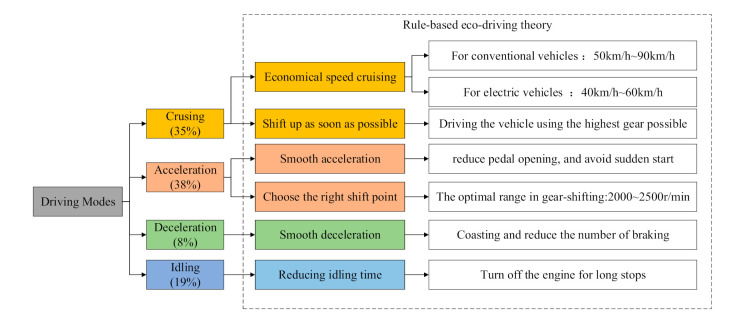
Rule-based eco-driving theory in the main driving modes.

Rule-based eco-driving theory refers to the energy-saving driving behavior summarized based on driving experience, road tests, or theoretical cognition. The process of driving the vehicle mainly includes the four driving modes of acceleration, deceleration, cruising, and idling [24,25]. Related research shows that under urban conditions, the energy consumption of the four driving modes accounts for 38%, 8%, 35%, and 19%, respectively [24]. The main energy-saving driving behaviors under various working conditions are shown in Figure 4. The main operations include economical speed driving, smooth driving, shifting up as soon as possible, and reducing idling time [26].

Economical speed cruising. For ICE vehicles, the driving speed affects the engine speed and load rate. Generally, the fuel consumption–driving speed curve is a concave curve, and there is a speed with the lowest fuel consumption, which is an economical speed. Driving at a stable speed can save a lot of fuel [27]. EI-Shawarby et al. [28] evaluated the impact of vehicle cruise speed on vehicle fuel consumption using data gathered under real-world driving conditions and their analysis showed that fuel consumption rates per unit distance are optimum in the range of 60–90 km/h. Wang et al. [29] collected vehicle operation data under congestion and normal conditions and demonstrated that the range of economic speed is between 50 and 70 km/h. Because the high-efficiency speed range of the motor is lower than that of the engine, the economic speed of EVs is generally lower than that of ICE vehicles [30]. Electric vehicle fuel consumption per unit distance is optimum at speeds between 40 and 60 km/h [31]. However, the economic speed is not fixed because it is affected by factors such as slope, weather, and load. Meanwhile, the speed of the vehicle should be affected by factors such as the traffic environment, driver preference, and the urgency of the driving task in actual driving. It is hard for the driver to choose a suitable economical speed and implement it.

Smooth driving. This refers to reducing hard acceleration and hard deceleration behavior so that the vehicle can smoothly transition between various working conditions. During aggressive acceleration, the fuel supply suddenly increases, resulting in insufficient fuel combustion and increasing fuel consumption [26]. The frequency and intensity of acceleration/deceleration lead to an increase in fuel consumption [29,32]. Wahlberg et al. [14] studied the relationship between acceleration and deceleration and fuel consumption by using linear regression based on measured data and showed that fuel consumption will decrease 2.8 L/km with every 0.1 m/s^2^ increase in deceleration, and 1.8 L/km with the same increase in mean acceleration. For urban driving vehicles, due to a large number of stop-and-go situations, reducing the intensity of acceleration and deceleration is the most effective for reducing energy consumption [33]. Chio et al. [34] studied the critical acceleration values that caused the fuel consumption of LPG passenger vehicles to increase sharply during parking acceleration and driving acceleration, which were 2.598 m/s^2^ and 1.4705 m/s^2^, respectively.

In addition, different from the smooth driving and constant speed cruise strategy, there is also a pulse-and-glide (PNG) dynamic cruise fuel-saving strategy. PNG means that the vehicle first accelerates from the low-speed phase to high-speed phase (acceleration phase), and then freely reduces from the high-speed phase to the low-speed phase (the coasting phase). The two phases form a complete PNG, where the average speed is equal to the desired speed [35]. This strategy was first studied by E. Gilbert, who theoretically deduced and compared the fuel-saving effects of “quasi-relaxed steady state (QRSS)” and “periodic control” and verified that the periodic control is better in cruise mode [36]. Researchers in recent years have shown that the PNG driving strategy also has good fuel-saving effects in hybrid and electric vehicles [37,38]. Automatic vehicles seem to be a good carrier for this driving strategy because it requires periodic control of power components, which is difficult for the driver to implement.

Shifting up as soon as possible. Shifting up as soon as possible increases the engine load rate and helps control the engine speed in the economic zone [26]. Beusen et al. [39,40] studied the gear information of drivers whose fuel consumption had been significantly reduced after eco-driving training and found that the average shift point changed significantly (moved closer to the optimal 2000 revolutions per minute (r/min)). Choosing the right shift point (generally, 2000 r/min–2500 r/min) and driving the vehicle using the highest gear possible are effective methods to reduce fuel consumption for conventional vehicles. However, as automatic transmissions become more and more popular in vehicles, this energy-saving driving strategy is losing its meaning.

Reducing idling time. Idling is a working condition in which the engine is idling but does not output power, resulting in fuel consumption. If the vehicle is idling for more than half a minute, the driver is recommended to turn off the engine, because the fuel required to restart the engine is less than the fuel consumed while the engine is idling. If the above practices are avoided, combined with other factors, the savings can be up to 20% [8]. An important reason why hybrid and electric vehicles have better fuel-saving effects than ICE vehicles is the elimination of idling conditions. For hybrid vehicles, the engine is directly controlled by the motor to start and stop, so the engine does not have an idling condition to reduce fuel consumption.

The rule-based eco-driving theory is derived from the summary of experiences or experiments and it has the advantages of strong guidance and fast fuel-saving effects. The training and research of eco-driving projects in many countries are carried out by teaching drivers this kind of rule-based driving theory [41,42]. However, because eco-driving theory is a qualitative description of energy-saving driving operation, the fuel-saving mechanism is not clear, and it is not the optimal solution in practical applications. In addition, this rule-based eco-driving strategy is a group-oriented eco-driving suggestion, and it cannot provide targeted suggestions on the driving behavior of individual drivers.

### 2.2. Optimization-Based Eco-Driving Theory

Optimization-based eco-driving theory refers to the optimal vehicle control strategy, which is solved through optimization algorithms based on the vehicle model combined with the current geographic or traffic information. Compared with the rule-based eco-driving theory, theoretically, it has the optimality of fuel-saving effects due to the optimal matching of driving behavior and traffic elements.

Gradient information is a very critical geographic factor in the study of eco-driving theory. Saerens et al. [43] designed predictive cruise control (PCC) considering the slope for heavy-duty trucks. Pontryagin’s maximum principle (PMP) was used to solve the optimal velocity profile because of its advantages of simple and fast calculation. This eco-driving strategy was able to save 5% of fuel, compared to a conventional cruise strategy with a reference velocity of 104 km/h. Erik et al. [44,45] added the restriction condition of not increasing the total travel time on the basis of the above research problem. The simulation results based on a dynamic programming algorithm (DP) and model predictive control (MPC) showed that the fuel consumption was reduced by 3.5% and 2.5%, respectively. If the total travel time was not limited, the fuel consumption was reduced by about 2–3% while the travel time increased slightly, by about 1.5% which means the maximum increase was 1 min for 1.5 h of travel time [46]. In addition to conventional cars, Chen et al. [47] proposed a predictive driver coaching (PDC) system for hybrid electric trucks that uses static map data and dynamic traffic data to achieve a trade-off between fuel consumption and trip time.

At present, in the eco-driving strategy, when considering the impact of slope on energy consumption, the optimization algorithm mainly adopts PMP [43,48,49,50,51], DP [44,46,52], and MPC [19,44]. The research objects are mainly heavy trucks and buses [43,44,45,46,47,48,49,50,51,52] because the impact of slope on energy consumption is significant for vehicles with a large mass. The above analysis shows that the impact of slope on this heavy vehicle is about 2.5% to 5%, which is relatively small compared to the impact of other factors on vehicle fuel economy. If the slope factor is considered in the study of small passenger cars, the improvement in energy consumption will be smaller, and it is easy to be masked by other factors.

In addition, with the development of machine learning, the field of eco-driving strategy has also begun to use intelligent algorithms to solve optimal control. Lee et al. [53] developed a model-based reinforcement learning algorithm (MBRL) that considers the road gradient for the eco-driving of electric vehicles. The simulation results indicated that the speed profile optimized using model-based reinforcement learning had similar behavior to the global solution obtained via DP and exhibited an energy-saving performance of 1.2–3.0%, which is similar to DP.

Traffic lights at intersections are the most common road traffic facilities on urban roads. Although ensuring the safety of traffic, it also harms the energy consumption of vehicles. The vehicles must decelerate to a complete stop, idle, and then accelerate to the required speed with traffic signal restrictions, which greatly increases the energy consumption and emissions of the vehicle. Speed trajectory guidance in the traffic signal light scene has great potential for energy saving [54]. Wu et al. [55] tried to provide information about the signal light to drivers to inform about whether the vehicle can pass the signal light, reducing the deceleration process of the vehicle to avoid wasting energy, but did not give specific driving operations. However, only considering reducing the deceleration process, there may be safety issues. Asadi et al. [56] used short-range radar to obtain the distance to the vehicle in front, combined with traffic signal information, and adjust the speed trajectory with the minimum braking amount and maintaining the safety distance between vehicles as the control objectives. A data-driven opportunity-constrained eco-driving control method [57] was proposed for speed trajectory planning under uncertain signal timing to improve the robustness of the optimal speed trajectory to random traffic light times. The results showed that it can significantly reduce fuel consumption while sacrificing less than 5% of the time cost.

Meanwhile, traffic information and geographic information should be considered comprehensively. Ozatay et al. [58] established a cloud platform where the driver inputs the destination into the server, generates a route, and collects surrounding traffic and geographic information, and solves the optimization problem by a spatial domain DP to obtain the vehicle speed curve and convey it to the driver. The results of the actual vehicle verification showed that the fuel economy improved by 12.6% on average for highway driving and 7.4% for urban driving. The difficulty for drivers to drive at a given speed trajectory due to the large external interference seems to be the main reason for the small improvement of fuel consumption in urban driving. Meanwhile, when making eco-driving recommendations, the driver’s driving preferences (such as longitudinal and lateral acceleration, vehicle spacing) should be considered to improve the driver’s acceptance of eco-driving recommendations [59,60]. Eco-driving operation recommendations should be the result of trading off driver preference and fuel economy.

**Figure 5:**
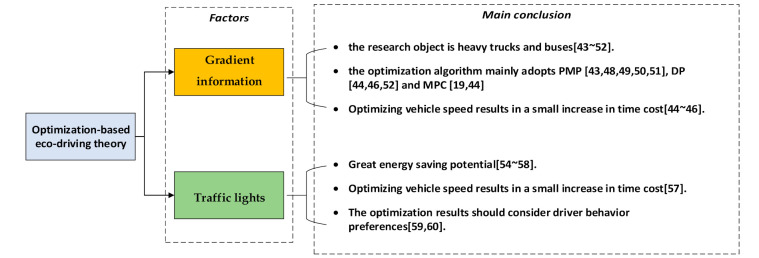
Main factors and conclusions of optimization-based eco-driving theory.

Figure 5 shows the main factors and conclusions in the ecological driving theory research. However, the above research was carried out on a single vehicle. Tielert et al. [12] compared the fuel consumption of a single vehicle under the signal light with the fuel consumption in the road network. Compared to the former’s 22% fuel economy improvement, the latter’s fuel consumption was reduced by only 8%. In fact, under actual complex traffic conditions, it is difficult to control a single vehicle to follow its optimal energy consumption speed trajectory. How to make a unified speed plan for the fleet or area to achieve the optimal energy consumption of the fleet [30] or area [61] is one of the future research directions for eco-driving theory. At the same time, studies on energy-saving driving strategies are carried out in single scenarios such as signal lights, cruise, and car following, and there is a lack of research on eco-driving theory that considers global optimization of all working condition information.

Previous research is limited by the difficulty in obtaining traffic information, and the above problems are difficult to solve. However, with the development of vehicle networks and big data, it seems to provide new ideas for solving this problem. For example, some researchers have proposed the concept of smart and connected cities (SCC) [62]. Private cars can plan travel routes and avoid traffic congestion by obtaining traffic congestion information in advance [62]; special-purpose vehicles can reasonably plan routes based on traffic information to ensure service levels and reduce energy consumption [63]. It is a challenge and an opportunity to use the information and data provided by the Internet of Vehicles and big data to realize eco-driving in all working conditions in the region.

## 3. Eco-Driving Capability Evaluation

Several studies have confirmed that unless people can see the beneficial consequences of certain behaviors in time, it is difficult for individuals to maintain such behaviors over time [64]. For many drivers, a small reduction in fuel budget caused by eco-driving behavior is difficult to detect, and it is not enough to trigger them to make some behavioral changes [33]. The lack of evaluation and feedback on eco-driving behavior is the root cause of drivers’ inability to persist [65]. In addition, transportation companies (such as bus operation companies, express transportation companies, etc.) encourage drivers to employ energy-saving driving through economic incentives, which also requires reasonable evaluation of drivers as a basis for reward. Therefore, it is of great significance to accurately evaluate driver eco-driving capability. This section reviews the ecological evaluation methods of driving behavior.

### 3.1. Qualitative Evaluation

Qualitative evaluation refers to the classification of drivers into different categories according to their energy-saving driving level, but the level of eco-driving of drivers in the same category is not distinguished. Driving style is one of the most obvious manifestations of the heterogeneity of driving behavior and is an effective way to qualitatively distinguish different levels of eco-driving through driving style.

Different driving styles have varying degrees of impact on energy consumption. Generally, a driver with an aggressive driving style consumes more energy than a driver with a mild driving style in the same situation [33,66]. Ericsson et al. [67] investigated the relationship between 62 basic driving parameters and driving style based on a large number of measured data. The results showed that 16 of them can reflect driving style well, and the relationship between those parameters and fuel consumption was elaborated. For vehicles powered by diesel engines, Rafael et al. [68] evaluated the effects of three driving styles on their fuel efficiency and emissions on a chassis dynamometer and the results showed that aggressive-style drivers have low fuel efficiency and high emissions. In addition, Fonseca et al. [69] proposed the Dynamic Performance Diesel Index (DPDI) formula, which is obtained by the linearly weighted accumulation of the vehicle gear position and average acceleration information and divides the driving style into economy, normal, and aggressive through the value of the DPDI. The most intuitive performance of driving style is acceleration and deceleration [68,70]. Drivers with aggressive driving styles tend to accelerate and decelerate rapidly. Since the second derivative of the vehicle speed characterizes the speed of the acceleration/deceleration change, it seems reasonable to reflect the driving style through the jerk, and Murphey et al. [71] found that the fuel consumption curve has a positive correlation with jerk.

Besides, hybrid and electric vehicles can recover energy when decelerating, so the driving style has a more obvious impact on energy consumption [72]. Bingham et al. [73] studied the influence of driving style on the energy consumption of electric vehicles based on vehicle speed, acceleration, and SOC. The results showed that the difference in energy consumption between smooth driving and aggressive driving can be up to 30%. However, it needs to be pointed out that different types and performances of vehicles affect the effect of driving style on energy consumption, but will not have a significant impact on the recognition of driving style; that is, the same person driving vehicles with different performances will not show different driving styles [74]. In fact, the boundaries between driving styles is not clear. It is reasonable to use fuzzy theory to study driving styles and energy-saving driving levels [75,76]. Select driving parameters (accelerator pedal position, acceleration, and deceleration) have a significant impact on energy consumption, and based on fuzzy theory, divide the driving style into energy-saving, good, qualified, and energy-consuming.

There is no doubt that driving style is related to vehicle energy consumption, and driving style can be used to characterize the level of eco-driving. However, it has been demonstrated that driving style is influenced by various factors such as the traffic flow and the road environment [77,78]. A driver may present different driving styles in different traffic environments. For example, a moderate-style driver will frequently accelerate and brake due to the restrictions of the congested traffic environment, and thus it is possible to present an aggressive and energy-intensive driving style. In addition, the driving style can only qualitatively distinguish between high-energy driving behavior and low-energy driving behavior, and cannot make a quantitative evaluation of the driver’s energy-saving driving level. If two drivers belong to the same type of driving style, it is difficult to evaluate the energy-saving driving level of the two drivers.

### 3.2. Quantitative Evaluation

Vehicle energy consumption is the most direct manifestation of a driver’s energy-saving driving level. In the past 10 years, extensive research has been conducted on the energy consumption modeling of conventional vehicles and electric vehicles [79]. It is necessary to clarify the quantitative relationship between vehicle operating parameters/driving behavior and energy consumption to estimate and predict vehicle energy consumption so as to better realize vehicle control strategies and traffic management strategies [9,44,45,46]. In addition, this relationship can also be used to guide the selection of evaluation indicators, and then use appropriate evaluation methods to evaluate eco-driving capabilities.

Some scholars have established different evaluation models with energy consumption as the evaluation index to evaluate the driver’s eco-driving capability. Xing et al. [79] proposed a personalized joint time-series modeling system to estimate the future energy consumption index. They thought that the energy consumption prediction index could be fed back to the driver to remind the driver of eco-driving. Liu et al. [80] used factor analysis to summarize the three characteristics into a comprehensive evaluation factor. The results showed that the correlation coefficient between the comprehensive evaluation factor and the fuel consumption of the vehicle was 0.9, and this was used as an indicator of the driver’s eco-driving capability. In addition, some models use data-driven methods to construct a non-linear relationship between energy consumption and driving behavior. Chen et al. [81] studied the relationship between driving behavior and vehicle energy consumption. Based on low-frequency data (0.05 Hz), the nonlinear relationship between driving behavior and vehicle energy consumption was fitted through a BP neural network. In his doctoral dissertation, Wu et al. [82] established eco-driving evaluation models for driving behavior and vehicle driving parameters based on the BP neural network. The input of those models are the parameters of driving behavior and vehicle driving and the output is the score converted into fuel consumption according to its set rules, which is used to quantify the ecological level of driving behavior.

In addition, eco-driving evaluation models have been established based on driving events [83,84]. Those models use the number of occurrences of certain driving events (such as rapid acceleration/deceleration) that affect the fuel consumption within a certain mileage as input and the output is the score that quantitatively evaluates the eco-driving capability. The results of this evaluation method are usually expressed in the form of scores. In terms of practical application, Suzhou Jinlong in China developed the G-BOS smart operation system [85]. This system uses nine indicators such as green zone driving (engine economic area) and long-term idling, sets the weight of each indicator, and scores driving behavior in the form of a 100-point system. However, this evaluation method requires expert knowledge, and when setting the evaluation weight coefficient, it is necessary to refer to the opinions of experts in the industry [84]. This increases the difficulty of model building and makes the model subjective.

In order to objectively evaluate the level of eco-driving, objective evaluation indicators are used to construct an eco-driving evaluation model. Zang et al. [86] evaluated eco-driving behavior based on vehicle specific power (VSP), and the differences between individual drivers’ VSP distributions and the baseline distributions were used to assess eco-driving behaviors. Zavalko et al. [87] proposed using the energy method to evaluate drivers’ economic driving level, and the energy consumed during braking could be used as an indicator to measure the eco-driving level. Based on the statistical logistic regression model, Andrieu et al. [88] proposed a calculation method of the eco-driving index with positive kinetic energy (PKE) as the parameter.

Quantitative evaluation methods can quantify the eco-driving level of a driver, but it still has its limitations. Table 1 summarizes the main eco-driving assessment methods and their limitations. Although the energy consumption is the most intuitive manifestation of eco-driving, it makes sense to use energy consumption to evaluate a driver’s level of eco-driving only when the vehicle type and road characteristics are controlled uniformly [18]. This is obvious, as even a very efficient driver will have a higher fuel consumption when driving under congestion than an inefficient driver driving on an unobstructed road. An efficient indicator of eco-driving should not depend on such external conditions and rely more on driver actions [88].

In fact, with the development of artificial intelligence technology, it has been applied in eco-driving evaluation and has solved some limitations of existing methods. In the evaluation method based on driving events, it is difficult to reasonably set the weights of various indicators. Kim et al. [89] used machine learning methods to determine the contribution of input features (driving events) in predicting target tags (fuel economy). Each driving event weight was allocated according to the machine learning contribution result. Han et al. [90] used unsupervised learning algorithms to mine energy-saving driving behavior in the historical data set and used the results as evaluation indicators. Kedar et al. [91] used principal component analysis (PCA) and clustering algorithms to directly evaluate ecological driving, which overcomes the subjectivity of the evaluation process. In addition, the Internet of Vehicles (V2I) provides a data basis for ecological driving evaluation, so that driving behavior can be evaluated more reasonably [92,93]. At present, artificial intelligence technology is mostly used for driving safety assessment, and the application of artificial intelligence technology in the field of eco-driving remains to be explored.

**Table 1:** Eco-driving evaluation methods and limitations.

Index	Methodology	Limitations
Driving style [34,66,67,68,69,70,71,72,73,74,75,76,77,78]	Input: vehicle operating/driving behavior dataOutput: economy, normal, aggressive	Cannot achieve quantitative evaluation. Evaluation result is affected by traffic factors.
Scoring [83,84,85]	Input: frequency of high-energy-consumption driving eventsOutput: eco-driving score	Requires expert knowledge and the model is subjective. Traffic factors impact results.
Fuel consumption [81,82,94,95,96,97]	Fuel consumption for a certain distance to measure eco-driving	Many factors affect vehicle energy consumption, and it is difficult to control other factors.
Others [86,87,88]	VSP, the energy consumed during braking, eco-driving index with PKE	Indicators are not intuitive. Traffic factors impact results.

## 4. Eco-Driving Application

Eco-driving is an energy-saving and emissions-reduction behavior that allows the public to participate, and it has great energy-saving potential. The development of energy-saving driving behavior in all of society is a complex task involving education, regulation, fiscal incentives, and social norms reinforcement [98]. The implementation of eco-driving is divided into antecedent intervention and consequence intervention from the behavior analysis. It is also referred to as static assistance and dynamic assistance. This section reviews the current implementation of eco-driving, the application effect, and the problems in the implementation process.

### 4.1. Antecedent Intervention

Antecedent intervention is eco-driving education and training, and it is the most direct way of eco-driving application, using text, video, or coach guidance to train drivers to guide them to gradually get rid of their bad driving behaviors and driving habits to achieve the purpose of energy saving. The knowledge of the training is the rule-based eco-driving strategy introduced earlier [27,28,29,30,31,32,33,34,35,36,37,38,39,40].

#### 4.1.1. Effectiveness

The earliest eco-driving education began in the early 1980s, mainly in Europe, Japan, Australia, and North America, and carried out the propaganda and education of ecological driving behavior [14,17,39,40,41,42]. The fuel-saving effect after eco-driving training has also been verified.

In an eco-driving training project carried out in Australia, a sample of 1056 private drivers were monitored for 7 months; 853 received eco-driving education, and 203 were monitored as a control group, and the study found that driver education led to a reduction in fuel use of 4.6% (0.51 L per 100 km) [95]. The Centre for Renewable Energy Sources (CRES) of Greece conducted an eco-driving pilot study [96]. Three bus drivers received theoretical training and their average fuel consumption on the scheduled 15 km route was reduced by 10.2%. A “JGSP Belgrade” eco-driving pilot program carried out in Serbia trained 13 bus drivers and reduced their fuel consumption by 8.61% in a short period of time [42]. Zovak et al. [97] compared the training effects after eco-driving education, and the results showed that fuel consumption and carbon dioxide were reduced by 32%. Although there are a few studies [14,41] that show that eco-driving training does not affect fuel consumption, since many factors (temperature, weather, participant’s personality) affect fuel consumption, it is possible that they affected the training effect.

#### 4.1.2. Effectiveness Factors

Eco-driving training has the effect of reducing energy consumption, but there are significant differences in the results of different studies on the specific impact on energy consumption. As mentioned above, the impact of eco-driving training on fuel consumption ranges from 4.6% to 32% [42,95,96,97]. The reason for this situation may be due to the differences in the drivers involved. For example, different drivers have different abilities to accept new knowledge, and before the training, the participants’ fuel-saving awareness is different. Beusen et al. [40] found that there are big differences in the impact of eco-driving education between different drivers, and 20% of driver participants did not achieve a reduction in fuel consumption. Meanwhile, the results of Barla et al. [94] also showed that drivers’ responses to eco-driving training were very uneven, with only about half of the participants achieving statistically significant fuel reductions. Due to the large number of people surveyed in the literature [95], the average fuel-saving effect may be reduced by some drivers. In addition to human factors, training methods and test conditions may also cause the above differences. Table 2 summarizes the factors that influence the effectiveness of eco-driving training and the main conclusions.

The driver’s previous cognition affects the effectiveness of eco-driving training. Valentina et al. [99] selected three drivers (extremely cost-aware, moderately cost-aware, extreme fuel consumer) to receive eco-driving training, and the average fuel consumption was reduced by 10.2%. The extreme fuel consumer reduced consumption by 20.4%, whereas the extremely cost-aware driver only reduced consumption by 4%. It can be seen that for drivers who are already energy efficient, the effect of eco-driving training is not obvious, and eco-driving training has a marginal effect. The research results of Stillwater et al. [100] also support the above view. He conducted a study on the cognitive precursors to driver behavior change and the results indicated that drivers that already had highly efficient driving styles did not benefit from eco-driving. Drivers who had low knowledge about fuel economy and low interest in fast driving had a significant improvement in fuel economy after training. Introducing eco-driving training for drivers who show a low level of eco-driving skill may be an efficient method for transportation companies to obtain economic benefits [87].

The driver’s driving experience and professionalism affects the effectiveness of eco-driving training. Wu et al. [101] measured the effectiveness of eco-driving training for both professional and non-professional drivers and they found that non-professional drivers showed a greater willingness to improve their behavior than professional drivers and the former had a more obvious improvement in fuel saving. Coloma et al. [102] studied the impact of eco-driving training on postal-truck drivers and got a similar conclusion. In fact, the essential difference between professional and non-professional drivers is the number of driving years and driving experience; thus, the reason for this difference may be related to the rich driving experience and long-term habits of professional drivers. It is difficult to change their driving habits only through short-term eco-driving training. It seems to be advisable to establish eco-driving policies in driving schools because novice drivers have not developed driving habits [103]. However, it should be pointed out that none of these studies considered the decline in driver comprehension due to age; professional drivers participating in the trial were usually older than non-professional drivers.

The form of eco-driving training affects the training effect. Andrieu et al. [104] analyzed two kinds of experiments. Simple advice was given to the participants in the first one, whereas in the second one, full courses with eco-driving experts were used. The fuel saving effect of the latter was better than the former. However, related studies have pointed out that purely theoretical eco-driving training is of no significance to energy saving, and actual training and coaches’ practical guidance are needed [41]. In addition to using traditional teaching methods (text, video teaching, coach guidance, etc.), Beloufa et al. [105] designed a specific guidance system interface that is embedded in a high-scale simulator and the drivers acquired eco-driving skills in an interactive, dynamic environment. Compared to traditional video learning, the interactive guidance system has a greater reduction. According to the current research, the best training effect seems to be immersive learning (practical exercises), followed by coaching in class, and the least effective is video and text education [101,104,105]. Although eco-driving training for drivers through a driving simulator has a good application effect [104,105,106], the cost is high and cannot be carried out on a large scale.

In addition, road traffic and weather also affect the effect of eco-driving training. The impact of weather on eco-driving is mainly reflected in that bad weather leads to bad driving conditions, and the driver’s requirements for driving safety are increased and the need for economy is suppressed, which leads to a decrease in the effect of eco-driving [107,108]. However, in terms of the impact of road traffic environment on the effect of eco-driving training, different scholars have reached different conclusions. Some scholars believe that driving on unobstructed roads is more conducive to the implementation of eco-driving strategies, so the fuel-saving effect is more obvious [102,109]. Coloma et al. [102] pointed out that eco-driving training seems to be more effective in small but not crowded cities. However, other scholars hold the opposite view [96,109]. Barla et al. [94] compared the average fuel consumption of cities and highways after eco-driving training, which was reduced by 4.6% and 2.9%, respectively. The results of the analysis clearly support that eco-driving training is more effective in congested urban environments. This conclusion also has a certain basis, because there are more opportunities to implement eco-driving behaviors on urban roads (acceleration/deceleration, idling).

#### 4.1.3. Limitations

Although eco-driving training has the advantages of quick results and low cost, it still has limitations. The biggest problem that limits its practical application is that the optimization effect of eco-driving training will gradually fade over time. Drivers’ driving behavior has a certain tendency, and it is very difficult to change driving habits. Even if it changes in a short amount of time due to external factors, when those external factors are eliminated, the effect will decline over time, and they will even return to their original driving habits [59]. Eco-driving training did indeed achieved good energy-saving effects in the short term, but the long-term effect is not good.

Barla et al. [94] found that the reductions faded gradually after the course. The fuel consumption reduction on urban roads was reduced by 4.6% to 2.5% within 10 months and highway reduction became statistically insignificant after 30 weeks. Wahlberg et al. [14] investigated the average fuel consumption over 12 months after eco-driving training and found that it was only reduced by 2%. This value can easily be masked by other factors. However, Zovak et al. [97] measured fuel consumption three months after eco-driving training. Compared with the measurement result immediately after the training, drivers’ fuel consumption had not changed much. This means the attenuation of the training effect after three months is not obvious. In addition, it seems that if there is follow-up evaluation feedback or non-economic incentives, the effect of eco-driving training can be prevented from declining [16]. Many studies have pointed out the decline of eco-driving training [14,16,39,40,41,94]. However, currently, there is little research on the question of how long the effect of eco-driving training will attenuate to a certain level. This is a big challenge. The long period, the difficulty in data collection, and the emergence of many uncontrollable factors are all problems that need to be faced. However, it does have research significance. For fleets or transportation companies, if eco-driving training is to have a long-term effect, it is necessary to conduct eco-driving training for drivers regularly [99]. It can be used to guide the development of training courses when the decay rate of the training effect is determined.

Overall, static assistance is necessary, although there are deficiencies in eco-driving education [109]. If eco-driving education can be carried out on a regular basis, it can play a very good role [111]. Meanwhile, it should be pointed out that knowledge education is unlikely to lead to permanent changes in driver behavior, because overcoming deep-rooted habits is not a simple matter; however, it can provide a theoretical basis and awareness for consequence intervention [98].

### 4.2. Consequence Intervention

Consequence intervention, which is also called dynamic assistance, refers to collecting and analyzing the driving behavior data of a driver through the in-vehicle device and feeding back information or actions to the driver to intervene in the driving behavior of that driver. This section mainly reviews the types, safety, and acceptance of in-vehicle feedback devices.

#### 4.2.1. Information Feedback

Information feedback during driving is a means to strengthen the driver’s awareness of fuel saving. The feedback information usually includes comprehensive ecological evaluation levels, instantaneous fuel consumption, and total fuel consumption. Only when the individual feels the bad influence brought about by the behavior in a timely manner will they be more proactive in changing that behavior.

In theory, this in-vehicle feedback device should have an impact on ecological driving, and the effect should be better than that of eco-driving training [14,108,112,113,114]. Seligman et al. [65] conducted research on the feedback effect on household electricity consumption. Through daily feedback to homeowners on electricity consumption, 5% more electricity consumption was saved than through pure education. For a Spanish garbage truck recycling eco-driving effect test project [112], 67 trucks were monitored for one year, and the average fuel saving with in-board driving-assistance devices was 7.45%. Eco-driving benefits obtained through real-time feedback did not tend to get lost over time because of an average difference of only –0.45% between the first and the last month of monitoring. Karlin et al. [114] conducted a meta-analysis of 42 feedback studies published from 1979 to 2010, and the results indicated that feedback is effective overall.

However, regarding whether the feedback is attenuating, some scholars have put forward different opinions [108,113,114,115,116]. Their test results showed that the effect of the feedback was the same as the eco-driving training. The feedback intervention time was negatively related to effects. However, perhaps after the introduction of a reward mechanism or game mechanism (gamification), the effect of feedback will be maintained or even increased [22,87,116] because of increased visibility and increased emotional and social components through competition. Madlen et al. [22] studied the impact of feedback, feedback plus gamification, and feedback plus gamification plus economic rewards on energy-saving effects for EV drivers. The results showed that gamification is more effective for pure feedback, and economic incentives did not add beyond the effects of non-reward-driven gamification that were already present. The combination of social incentives (non-economic) and feedback seems to be a good approach.

The form of information feedback mainly includes visual, auditory, or a combination of both [117]. Information about ecological evaluation, total fuel consumption, or instantaneous fuel consumption is presented to the driver through the dashboard or the panel and transmitted to the driver through a visual format. This kind of eco-driving feedback integrated into the vehicle dashboard is an effective method. At present, vehicle manufacturers have developed systems for eco-driving feedback [35,117]. In 2009, Honda developed a speedometer that can change color according to the driver’s eco-driving performance. Nissan has added an “ECO-P” light on the dashboard. When the driver has bad driving behavior, the light will turn yellow and flash. Others include, for example, Mercedes-Benz’s ECO Display system, BMW’s Eco Pro, etc.

However, while driving, the driver’s attention should be on the driving task. It is a problem that should be studied to convey eco-driving information through a simple and clear interface design because choosing the right graphics and color combination can better promote the driver’s interaction. The effective interaction of on-board systems, of course, involves ergonomics [118]. Using voice prompts, it seems safer to provide feedback to the driver from the auditory channel than the visual channel, because they do not need to remove their sight from the road, and it has also been confirmed that it has an impact on ecological driving [33,117,119]. It needs to be pointed out that there are two ways to convey information to the driver through auditory channels. One is to directly broadcast operational information. Drivers could get voice prompts (for example, “Please turn off the engine”) once non-eco-driving behavior has appeared [119]. The other is to convey information through tones, such as a predominantly low-frequency tone indicating insufficient accelerator depression and a high-frequency chime indicating excessive accelerator depression [117]. The comparison of the main methods of feedback for eco-driving and the advantages and disadvantages of different feedback methods are presented in Table 3. The combined audio-visual approach seems to be better than visual or auditory feedback alone.

Providing information feedback to the driver through visual or auditory means to improve eco-driving ability can have a good effect, but the real-time feedback method will take up some attention resources after all, and there may be safety and user acceptance issues (this issue will be discussed below). Offline feedback seems to be a good way to weigh the feedback effect and safety, with the assessment of the driver’s energy-saving driving level presented to the driver in the form of a report after the driver’s trip. Generally, this report should include the eco-driving level score, existing bad driving behavior, and suggestions for improvement. Zhao et al. [119] compared the energy-saving effects of the two feedback methods: visual–auditory and offline feedback. Compared to the 5.45% fuel consumption reduction caused by the visual–auditory feedback, the fuel consumption reduction effect caused by offline feedback was 3.43%. Although the fuel-saving effect was not as good as the visual–auditory feedback, they found that the offline feedback method was more acceptable. Safety is also an advantage of offline feedback. In addition, offline results can also be used as a basis for fleets or companies to reward drivers for economic driving [120,127].

#### 4.2.2. Action Feedback

Action feedback is transmitting information to the driver through a haptic form to warn the driver of bad driving behavior, which is real-time feedback. At present, haptic feedback for the purpose of fuel economy is mainly achieved through the pedal (as showed in Table 3, including force feedback, stiffness feedback, and vibration feedback). In addition, haptic reminders for safety (avoiding collision, lane departure) are realized through the steering wheel or seat [123]. In terms of practical applications, Nissan and Continental have respectively launched “eco-pedal” technology [121] and active gas pedal technology [124], which can remind drivers of economical driving by applying reaction force to the pedal or generating pulsed beating. Developers claim that it can help save fuel by 5–10% and up to 7%, respectively. In addition, Brirell et al. [123] designed an energy-saving driving device based on haptic feedback. When the throttle exceeds 50% (this threshold is considered non-economic driving), force feedback is performed to remind the driver to reduce the throttle. In the actual driving process, the driver’s visual burden is already very heavy, and it is a good feedback method to transmit information to the driver through a haptic form. Compared to visual feedback, the driver seems to rely more on haptic feedback [106].

**Figure 6:**
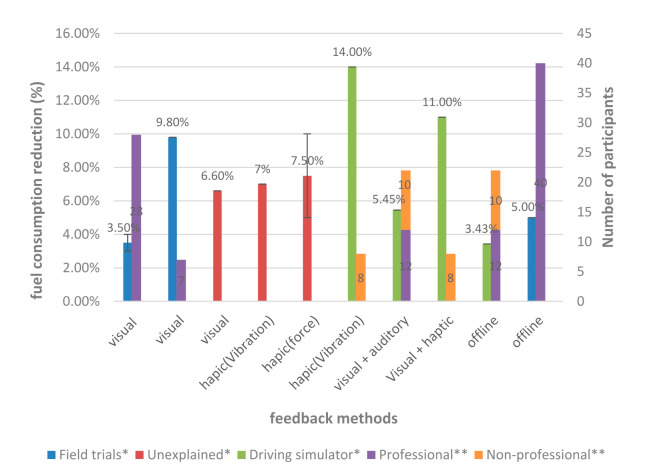
The effect of feedback method and test scale on energy saving. Data acquisition methods are divided into three categories, among which “Unexplained” represents that the acquisition method is not given in the study. Error bars represent the maximum and minimum values. Symbols for this figure: * fuel consumption reduction, ** number of participants. If the bar is not shown it means it is not stated in the study. economic incentives: [14,108,115,116,117,118,119,120,121,122,123,124,126,127].

The energy-saving effects of the main feedback methods are compared in Figure 6. It should be pointed out that the ordinate represents the energy-saving effect of different feedback methods. Although the test methods (field test or driving simulator) and the number of people used in different articles were different, the energy-saving effect was obtained by setting the control group and the experimental group in the cited article. Therefore, the influence of different scenarios on the result was eliminated. It can be seen in Figure 6 the effect of haptic feedback on eco-driving seemed to be better than visual feedback. This may have been due to the heavier visual burden causing the driver to pay less attention to the effect of visual feedback, and haptic feedback may have caused unconscious actions of the driver [125]. However, acceleration is not the only factor that affects energy consumption. By controlling only the accelerator pedal opening, the fuel saving potential is limited [115].

#### 4.2.3. Safety and Acceptance

Since eco-driving was put forward, the issue of its safety has been discussed. Regarding eco-driving training, people have raised safety questions mainly from the perspective of eco-driving strategy, and real-time feedback from in-vehicle devices is thought to be a distraction for drivers [128].

Generally, the fuel-saving strategy of eco-driving mainly includes driving at an economical speed, reducing rapid acceleration or rapid deceleration. Slight acceleration poses a safety risk during lane changing and reducing braking to improve the fuel economy may result in too small of a headway. However, several studies have shown that after eco-driving training, the accident rate decreased, which has no effect on safety [14,129]. In fact, when faced with the choice between safety and economy, out of instinct, the driver will definitely choose safety. The impact of eco-driving on drivers is often only reflected in the premise of sufficient safety, so safety and ecology do not conflict here [130].

The impact of real-time feedback on safety really needs to be studied, because it is undeniable that it does distract the driver. However, according to the current research results, in-vehicle feedback devices do not affect safety [23,105,131,132]. Rouzikhah et al. [131] did a study on driver distraction based on a driving simulator, and set up three tasks: reading eco-driving information, changing a CD, and entering a five-digit number in the navigation system. The result showed that reading eco-driving messages presented a distraction risk for drivers, but the risk was far inferior to the tasks that involved physical and cognitive demands. Meanwhile, as the driver’s familiarity with in-vehicle feedback devices increases, this effect may be smaller. However, it should be pointed out that the above conclusions are the results obtained in the laboratory. Although the driving simulator can reproduce the actual driving environment well, the participants know that the simulator is virtual and it will not cause loss to themselves if an accident occurs. So, participants are relaxed and the influence of the feedback on the distraction effect is weakened. The impact on safety remains to be further studied, considering the driver’s familiarity with feedback devices in real scenarios.

Choosing an appropriate feedback method [106,117,119], designing an ergonomic interface [118,121], gamification [22,98,116], and economic incentives [120,127] are effective methods to improve the acceptance and effectiveness of in-vehicle feedback devices. In addition, appropriate feedback temporal granularity and feedback frequency could improve drivers’ acceptance and motivation. Fine-grained (reacting to instantaneous operation behavior) and high-frequency feedback can achieve better optimization results, but they may cause irritability and resistance to the driver, whereas coarse and low-frequency feedback may miss the improvement of fuel economy opportunity [112,116].

## 5. Conclusions and Outlook

Eco-driving has the characteristics of great energy-saving potential, quick short-term results, and low cost. It is an effective means to realize energy-saving and emission reduction in the transportation industry in the short term. This paper reviews and summarizes the main literature in the field of eco-driving from the three progressive levels of theory–evaluation–application. Under such a framework, the main research in the field of ecological driving can be well summarized:

- Eco-driving theory. This is a theoretical study of driving behavior that reduces vehicle energy consumption. According to different sources, eco-driving theory is divided into two parts: rule based and optimization based. Rule-based eco-driving theory is qualitative and group-oriented guidance. Although it is easy for drivers to implement, the energy-saving effect of its practical application is affected by the traffic environment and the characteristics of the driver. Optimization-based eco-driving theory refers to the current optimal driving behavior obtained through optimization algorithms based on the current traffic and geographic information of the vehicle, which theoretically has the best energy-saving effect.
- Eco-driving evaluation. This part is to study how to fairly and reasonably evaluate drivers’ eco-driving capability. The paper divides the evaluation methods into qualitative evaluation and quantitative evaluation. Driving style recognition is the most common method in qualitative evaluation. In quantitative evaluation, in addition to using energy consumption as the evaluation index, there are also scoring methods oriented around driving events and some objective indicators. However, whether it is a qualitative or quantitative evaluation method, a reasonable evaluation index should only depend on the driver’s driving behavior and cannot be affected by external conditions (for example, traffic environment, weather).
- Eco-driving application. This part is the practical application of eco-driving theory and eco-driving evaluation. Application forms include eco-driving training and in-vehicle feedback devices. Although the former is simple to implement, the training effect is affected by many factors and will fade over time. The latter can transmit information to the driver through visual, auditory, or haptic channels, but safety and acceptance issues should be considered.

Research on eco-driving has achieved many exciting results. However, it still has limitations. The following summarizes the shortcomings of the current three aspects of eco-driving theory, evaluation, and application. Meanwhile, combined with the current popularity of big data applications, the development of the Internet of Vehicles (V2I), and cloud computing platform technology, it has considered how to solve the above limitations:

- Currently, the study of vehicle speed trajectory planning with the goal of optimal fuel economy mainly focuses on a single vehicle, and rarely considers the energy consumption and emissions of the fleet or area at the network level. Mixed traffic flow should be considered when solving the optimal problem of regional energy consumption and emissions. In addition, research on energy-saving driving strategies is mostly carried out in single scenarios such as traffic lights, ramps, and car following, and there is a lack of global optimal eco-driving strategies that consider the information of all working conditions.

The development of the Internet of Vehicles technology (V2I) provides an information foundation for solving this problem. All vehicle information and traffic road information in the area can be obtained through V2I. By combining the above information, the vehicle route and speed in the area can be planned based on the cloud computing platform to achieve optimal energy consumption in the area. Meanwhile, accurate energy consumption models are the basis for carrying out theoretical research on eco-driving based on optimization and vehicle operation. Big data provides a data basis for using data-driven methods to build a highly non-linear relationship between driving behavior and energy consumption.

- Driving style can only qualitatively evaluate eco-driving capability, and quantitative methods (such as scoring) require expert knowledge. In the existing research, the important parameters used to evaluate eco-driving are speed, acceleration, and energy consumption, among which energy consumption is a broad and intuitive indicator. However, it is very difficult to explain that energy consumption is only caused by different driving behaviors because many factors affect the final energy consumption of a vehicle (weather, traffic, load, etc.). Only when the driver is in the same driving condition does it make sense to use energy consumption to compare the driver’s energy-saving driving level. Research in this area is often carried out under experimental conditions (such as driving simulators [105,106]) in order to control the impact of other variables on energy consumption to verify the application effects of training or vehicle feedback devices, but the actual application effects are difficult to verify.

With the application of big data technology to vehicles, the abundant driving conditions and massive vehicle operating data provide a data basis for solving the standardization of variables. The historical big data is divided into groups according to driving conditions (weather, traffic, road geometry, etc.) and extract energy consumption indicators for each group. Since the eco-driving evaluation is based on energy consumption indicators with similar driving conditions, it can exclude the influence of external factors, making the evaluation results more reasonable.

- The attenuation characteristics of the energy-saving effects of eco-driving training under long-term conditions are unclear, and there is no unified conclusion about whether the effect of in-vehicle feedback devices fades over time. The research in this area is mainly limited by the long test period, the difficulty of data collection, and the small number of sample data. Fortunately, big data of vehicle operation over a long period have made it possible to study the effect of eco-driving training and in-vehicle devices under the influence of a long period of time.

## Figures and Tables

**Table 2:** Factors that influence the effectiveness of eco-driving training and conclusions.

Factors	References	Main Conclusion
Effectiveness ^1^	[39,42,94,95,96,97,98]	Improves fuel economy immediately. Fuel saving ranges from about 4% to 32%. Highly heterogeneous between individuals Theoretical eco-driving training has no effect.
	[14,41]	
Previous cognition	[85,99,100]	Highly efficient drivers do not benefit much from eco-driving training. Driver with low interest in fast driving have significant improvement.
Profession/Driving experience	[101,102,103]	Non-professional drivers have a more obvious improvement in fuel saving than professional drivers.
Training form	[41,101,104,105,106]	Training effect from good to bad: immersive learning, coaching, video, and text education
Weather	[15,107,108]	Rain and snow affect the effectiveness because of the requirements for safety. Temperature affects the training effect.
Traffic environment ^2^	[102,109]	More effective on unobstructed roads More effective on congested urban roads
	[94,110]	
Recession	[14,16,39,40,41,59,94]	Declining over time

^1^ Scholars have the opposite view and conclusions on the effectiveness. ^2^ Scholars have the opposite view and conclusions on the traffic environment.

**Table 3:** The comparison of the main methods of feedback for eco-driving.

Method	Implementation	Advantage	Disadvantage
Visual	Displaying eco-driving level, fuel consumption, and suggestions through the dashboard/APP [14,113,116,118,120,121]	Clear and intuitive Can be post-installed Non-aggressive and high acceptance	Increases visual burden Distracting and not safe The effect depends on the understanding of the drivers.
Auditory	Delivering information through announcements [119] or tones [117]	Driver’s sight need not leave the road and high safety Effective at encouraging compliance	Intrusive interference and irritates the driver Low acceptance The effect depends on the understanding of the driver.
Haptic	Stiffness[122], Vibration [123,124,125], or force [115,117,121] feedback via pedal	Less distraction The driver reacts quickly Good execution effect Perceived only by the driver	Cannot be post-installed Intrusive interference and low acceptance Physical burden Feeling of uncontrollable vehicle
Combined	Visual + auditory feedback [112,117,118,122]	Reduced distraction Adds benefit to visual displays	Increase in workload Overlapping effects
	Visual + haptic feedback [106,122,126]	Fosters greater compliance Reduced distraction	
Offline	Feedback in the form of a report after the end of the trip [108,119,120]	No need to add equipment High safety High acceptance	No quantitative guidance Less effective compared to other methods
Gamification	Showing the level of eco-driving in the form of ranking [22,98,116]	No need to add equipment No distraction to the driver	Limited number of influences

## Data Availability

Not applicable.
